# Coronary plaque burden and computed tomography-derived fractional flow reserve are predictors of 5-year mortality in peripheral artery disease patients with no known coronary artery disease

**DOI:** 10.1016/j.jvscit.2026.102361

**Published:** 2026-07-27

**Authors:** Dainis Krievins, Sanda Jegere, Frank Arko, Aigars Lacis, Edgars Zellans, Gustavs Latkovskis, Indulis Kumsars, Davis Putrins, Janis Vetra, Edgars Supols, Ligita Zvaigzne, Lilian Tzivian, Linards Lacis, Andrejs Erglis, Christopher K. Zarins

**Affiliations:** a Department of Vascular Surgery, Pauls Stradins Clinical University Hospital, Riga, Latvia; b Faculty of Medicine and Life Sciences, University of Latvia, Riga, Latvia; c Sanger Heart and Vascular Institute, Charlotte, NC; d Faculty of Medicine, Riga Stradins University, Riga, Latvia; e Stanford University Medical Center, Stanford, CA

**Keywords:** Coronary CT angiography, Quantitative plaque analysis, CT-derived FFR, Silent coronary ischemia, Peripheral artery disease, Long-term mortality

## Abstract

**Objective:**

Patients with peripheral artery disease (PAD) have poor long-term survival due to coexisting coronary artery disease (CAD), which is often asymptomatic and undiagnosed. Coronary computed tomography angiography (CTA)-derived quantitative plaque analysis (CT-QPA) and fractional flow reserve (FFR_CT_) are established predictors of cardiovascular death in patients with symptomatic CAD. However, their prognostic value in patients with PAD with no symptoms of CAD undergoing peripheral vascular surgery is unknown. This study assessed the value of CT-QPA and FFR_CT_ in predicting 5-year mortality in patients with vascular surgery with no known CAD.

**Methods:**

Patients with PAD with no evidence of CAD who were cleared for elective peripheral vascular surgery were enrolled in a prospective study of preoperative CTA and FFR_CT_ to evaluate cardiac risk. Lesion-specific coronary ischemia was defined as FFR_CT_ ≤ 0.80, with severe ischemia defined as FFR_CT_ < 0.75. CT-QPA analysis provided quantitative data on total plaque volume (TPV) and plaque characteristics. Vascular surgery was performed on all patients with no perioperative mortality. Postoperatively, all patients received guideline-directed medical therapy with no elective coronary revascularization. The primary endpoint was all-cause death at 5 years, assessed by Cox regression analysis.

**Results:**

Among 124 patients with PAD with no cardiac history or symptoms (mean age, 67 ± 8 years; 76% men), coronary plaque burden was high (median [TPV] 550 mm^3^ [IQR, 299-1156]), and 53% had asymptomatic (silent) coronary ischemia (FFR_CT_ ≤ 0.80). During a 5-year follow-up, 32 patients (26%) died. Nonsurvivors had nearly twofold higher TPV compared with survivors (975 vs 458 mm^3^; *P* < .001), with significant increases in calcified (*P* < .001), noncalcified (*P* < .001), and low-attenuation plaque (*P* = .038). Five-year mortality in patients with TPV > 750 mm^3^ was 46% compared to 4% in those with TPV < 250 mm^3^ (log-rank *P* < .001). Both TPV ≥ 550 mm^3^ (hazard ratio, 3.6; 95% confidence interval, 1.6-7.9; *P* = .002) and FFR_CT_ ≤ 0.80 (hazard ratio, 2.1; 95% confidence interval, 1.0-4.5; *P* = .045) were predictors of increased mortality risk. Five-year mortality in patients with both high plaque and coronary ischemia was 42% compared with only 10% in patients with low plaque and no ischemia (log-rank *P* = .006).

**Conclusions:**

In patients with PAD with no known CAD undergoing elective vascular surgery, coronary plaque burden and silent coronary ischemia detected by CT-QPA and FFR_CT_ were predictors of a 5-year mortality. Coronary CTA with quantitative plaque and FFR analysis should be considered for comprehensive cardiac risk stratification in patients with vascular surgery, regardless of cardiac symptoms.

**Article Highlights**

• **Type of Research:** Observational cohort study of patients with peripheral artery disease with no cardiac symptoms undergoing elective peripheral vascular surgery
• **Key Findings:** Systematic cardiac risk assessment using coronary computed tomography angiography (CTA) with quantitative plaque analysis and fractional flow reserve (FFR_CT_) revealed a high burden of coronary plaque and a high prevalence of silent (asymptomatic) coronary ischemia, placing patients at risk for cardiac death and myocardial infarction. Both coronary plaque burden and lesion-specific coronary ischemia were predictors of a 5-year mortality, with a dose-response relationship between total plaque volume and long-term mortality. Patients with both high-plaque volume and silent coronary ischemia had a 42% 5-year mortality compared with 10% in patients with low-plaque volume and no coronary ischemia (*P* = .006).
• **Take Home Message:** The long-term survival of patients with vascular surgery with no known coronary artery disease can be predicted by noninvasive cardiac evaluation using coronary CTA with quantitative plaque and FFR_CT_ analysis.

Patients with peripheral artery disease (PAD) undergoing vascular surgery procedures have poor long-term survival, with 50% to 55% 5-year mortality following open or endovascular lower-extremity revascularization.^1^^,^^2^ It is well-known that the primary cause of death is coronary artery disease (CAD), and that most patients have anatomic and physiologic evidence of significant CAD, which is frequently asymptomatic and undiagnosed.^3^^,^^4^ Current PAD and noncardiac surgery guidelines highlight the high cardiac risk but recommend no routine preoperative cardiac testing of patients without cardiac symptoms, relying instead on best medical therapy and risk-factor control.^5^^,^^6^ However, this strategy has not improved long-term survival with annual mortality rates following major vascular surgery essentially unchanged for the past four decades despite marked advances in medical and interventional therapy.^1^^,^^2^^,^^7^, ^8^, ^9^

In contrast, contemporary CAD guidelines recommend coronary computed tomography angiography (CTA) as a frontline diagnostic test for patients with suspected CAD and emphasize the use of plaque analysis and CT-derived fractional flow reserve (FFR_CT_) for risk stratification and treatment guidance.^10^, ^11^, ^12^ Coronary CTA with quantitative plaque analysis (CT-QPA) and FFR_CT_ provide quantitative measures of plaque burden and lesion-specific ischemia that have been shown to be strong independent predictors of cardiovascular death and myocardial infarction (MI) in symptomatic CAD populations.^13^, ^14^, ^15^, ^16^, ^17^

In 2017, we initiated a prospective study of systematic cardiac risk assessment using CTA and FFR_CT_ in patients with PAD with no cardiac history or symptoms undergoing major vascular surgery.^4^ Patients were evaluated with coronary CTA and FFR_CT_ to determine the prevalence of silent coronary ischemia and to assess the usefulness of this information in guiding patient care. This study demonstrated that patients with PAD had a high prevalence of silent coronary ischemia (about two-thirds of patients) and showed that ischemia-guided coronary revascularization following lower-extremity revascularization reduced death and MI by more than 50% compared with guideline-directed care alone.^18^, ^19^, ^20^ QPA was not yet available during the time of enrollment, so the prognostic value of plaque burden could not be assessed.

The present study focuses on the subset of patients from that program who were managed with guideline-directed medical therapy alone and did not undergo coronary revascularization, thereby allowing assessment of the natural history of asymptomatic CAD in a PAD cohort considered to be low risk for surgery. In this study, we used CT-QPA to quantify coronary plaque burden, and combined this with FFR_CT_-based assessment of lesion-specific coronary ischemia to evaluate their long-term prognosis following surgery. We hypothesized that quantitative plaque burden together with physiological coronary ischemia would identify patients at both high and low risk for 5-year mortality.

## Methods

### Study design and patient population

Patients for this study were drawn from a large prospective observational study of cardiac risk assessment using coronary CTA and FFR_CT_ in patients with PAD with no known CAD undergoing peripheral vascular surgery. The study was conducted at the Pauls Stradins Clinical University Hospital, Riga, Latvia, and represented the first systematic application of FFR_CT_ analysis in patients with PAD. Patients were enrolled from 2017 to 2019 at a time when the value of ischemia-guided coronary revascularization in patients with PAD was unknown and QPAs had not yet been developed.

Details of the study design and patient inclusion/exclusion criteria have been published^4^^,^^18^ and are briefly summarized here. Patients with PAD, aged ≥50 years with no cardiac history or symptoms who were admitted to the hospital and cleared for elective vascular surgery, were candidates for the study. Exclusion criteria included ischemic ECG abnormalities, previous MI or coronary revascularization, chest pain or suspected acute coronary syndrome, severe arrhythmia, pacemaker, renal insufficiency (creatinine > 130 μmol/L), contraindication to coronary CTA, and significant comorbidity with life expectancy <1 year. Patients enrolled in the study were evaluated with coronary CTA before the scheduled vascular surgery procedure. Coronary CT scans were sent for off-site FFR_CT_ analysis, and results were returned within 24 hours. However, downstream patient management, including diagnostic angiography and coronary revascularization, was not prespecified but was left to physician discretion. The protocol was approved by the institutional ethics committee, and all patients gave written informed consent.

When coronary CT-QPA became available in 2024 we reviewed the study database and selected all patients with FFR_CT_ analysis who had undergone lower-extremity revascularization or aortic aneurysm repair and were treated with guideline-directed medical therapy with no elective coronary revascularization. A total of 124 patients were identified and included in this study. The identifiers for CTA scans obtained from 2017 to 2019 were submitted to HeartFlow for CT-QPA analysis, with blinding to patient characteristics, treatment, and outcome. This allowed unbiased assessment of the natural history and prognostic value of plaque burden and ischemia in patients with PAD with no cardiac symptoms managed according to current guidelines.

### Coronary CTA imaging

Coronary CTA was performed using a single-source 64-row CT scanner with beta blockade for heart rate control and sublingual nitroglycerin for coronary vasodilation, in accordance with the Society of Cardiovascular Computed Tomography guidelines.^21^ CTA datasets were transmitted via secure web-based interface for computational analysis of FFR_CT_ (HeartFlow, Inc), with blinding of patient-specific clinical information. FFR_CT_ results were returned within 24 hours for physician interpretation and were available to treating physicians.

### Coronary ischemia assessment: FFR_CT_

**Fig 1:**
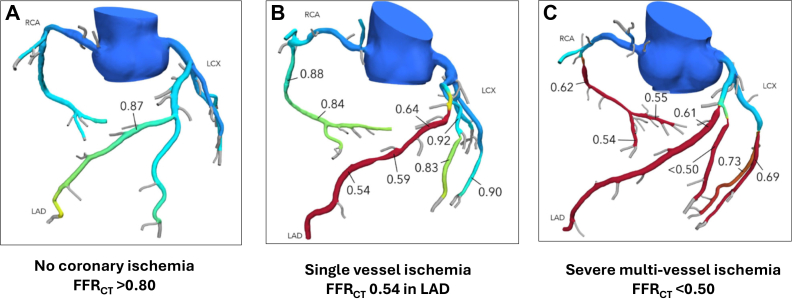
Coronary ischemia assessment using computed tomography (CT)-derived fractional flow reserve (*FFR*_*CT*_). Lesion-specific coronary ischemia was defined as FFR_CT_ ≤ 0.80 distal to >30% computed tomography angiography (CTA) stenosis with FFR_CT_ ≤ 0.75 indicating severe ischemia. No ischemia **(A)** was found in 47% of patients (FFR_CT_ > 0.80). Silent (asymptomatic) single-vessel **(B)** or multivessel **(C)** ischemia was observed in 53% of patients with peripheral artery disease (PAD).

FFR_CT_ analysis produced a three-dimensional color-coded model of the coronary arteries with FFR values throughout the coronary tree. Lesion-specific coronary ischemia was defined as FFR_CT_ ≤ 0.80 measured at 2 cm distal to a coronary stenosis ≥ 30% in vessels ≥ 2 mm diameter, and severe ischemia was defined as FFR_CT_ ≤ 0.75. For each patient, the severity of ischemia was determined from the lowest lesion-specific FFR_CT_ value. Examples of FFR_CT_ analyses demonstrating the presence or absence of lesion-specific coronary ischemia are shown in Fig 1.

### CT-derived quantitative plaque analysis

**Fig 2:**
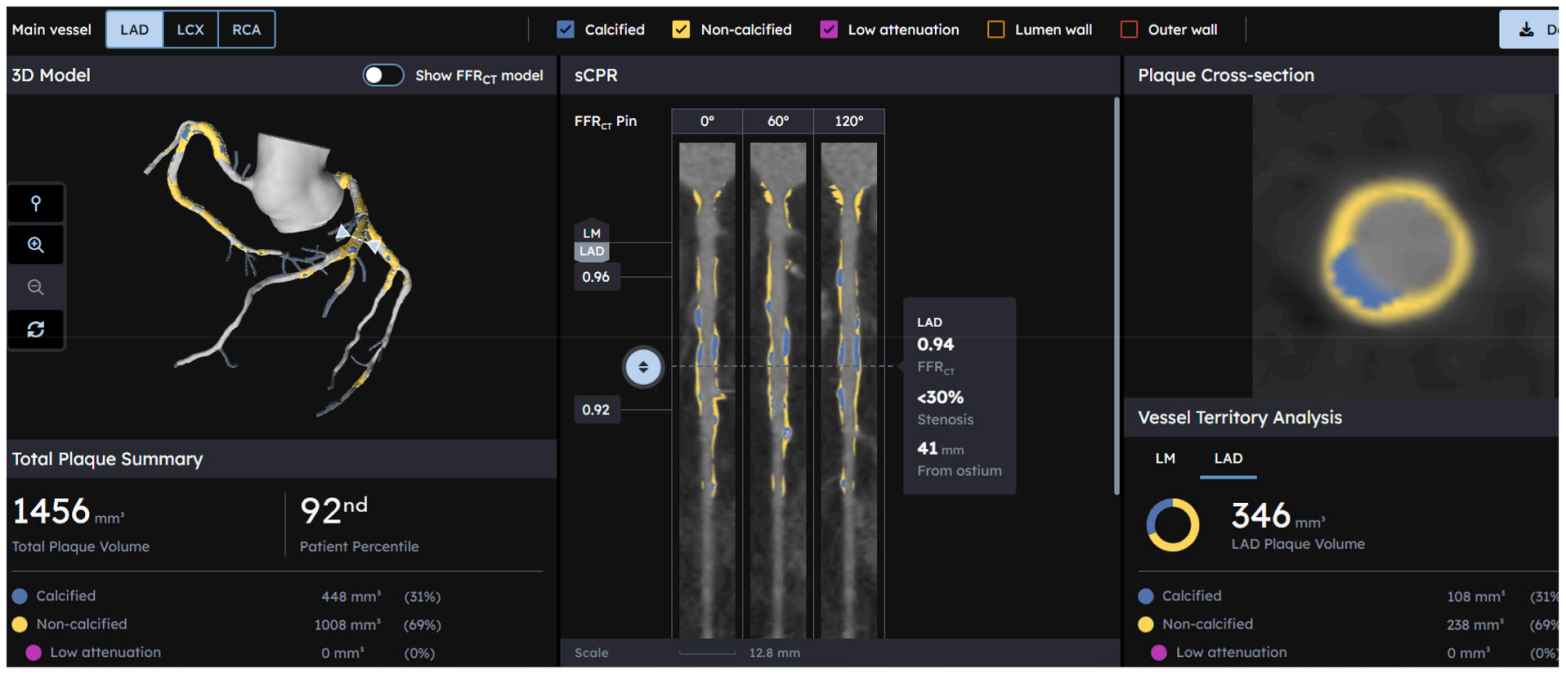
Coronary computed tomography (CT)-derived quantitative plaque analysis (*CT-QPA*). Case example of asymptomatic patient with very high total plaque volume (1456 mm^3^) comprised of noncalcified plaque (1008 mm^3^) and calcified plaque (448 mm^3^) and no evidence of coronary ischemia [CT-derived fractional flow reserve (FFR_CT_) > 0.80 throughout]. Dynamic controls enable interrogating the three-dimensional model and scrolling through each vessel with cross-sections and FFR_CT_ values at any selected site.

CT-QPA was performed on the original CTA datasets obtained from 2017 to 2019 in all 124 patients. The analysis quantified total plaque volume (TPV), calcified plaque volume (CPV; ≥350 Hounsfield units [HU]), non-CPV (NCPV; 30-350 HU), and low-attenuation plaque volume (LAPV; <30 HU). Patients were stratified by TPV using the median (550 mm^3^) and into three plaque burden categories: mild TPV ≤ 250 mm^3^, moderate 251 to 750 mm^3^, and severe > 750 mm^3^, consistent with published plaque severity staging.^22^ A case example of severe coronary plaque burden in a patient with no lesion-specific coronary ischemia is shown in Fig 2.

### Vascular surgery and postoperative management

Elective vascular surgery was performed as planned in all patients, with no perioperative (30-day) deaths. Lower-extremity revascularization accounted for 92% of procedures and abdominal aortic aneurysm repair for 8%. Following surgery, all patients were managed in accordance with the 2016 European Guidelines on Cardiovascular Disease Prevention in Clinical Practice.^23^ This included risk-factor modification with lifestyle changes in diet and exercise, weight loss and smoking cessation together with evidence-based medical therapy, including high-intensity statins, antiplatelet and/or anticoagulant therapy, antihypertensive agents, and glycemic control. No patients underwent elective coronary revascularization during the 5-year follow-up period.

### Five-year follow-up and endpoint

Long-term follow-up data (such as all-cause mortality and cause of death) were obtained from national and institutional sources, including the State Register of Natural Persons, hospital electronic records, medical records, and family physician or family reports. Follow-up was complete in all patients through 5 years. The primary endpoint was all-cause death at 5 years.

### Statistical analysis

Continuous variables were tested for normality with the Shapiro-Wilk test and reported as mean ± standard deviation or median (interquartile range) as appropriate. Categorical variables were expressed as counts and percentages. Patients were stratified by TPV (≥550 vs <550 mm^3^), plaque burden categories (≤250, 251-750, and >750 mm^3^), and presence or absence of lesion-specific ischemia (FFR_CT_ ≤0.80 vs >0.80). Group differences were evaluated with Student *t*-test or Mann-Whitney *U* test for continuous variables and χ^2^ or Fisher exact test for categorical variables. Survival curves were generated with the Kaplan-Meier method and compared using the log-rank test. Cox proportional hazards regression was used to estimate univariate and age-adjusted multivariate hazard ratios (HRs) and 95% confidence intervals (CIs) for predictors of 5-year mortality. Analyses were performed with SPSS Statistics version 29.0 (IBM Corp.). A two-sided *P* < .05 was considered statistically significant.

## Results

### Baseline patient characteristics and five-year mortality

Baseline characteristics of the 124 patients with PAD with no cardiac history or symptoms who were evaluated with CT-QPA and FFR_CT_ are shown in Table I. The mean age was 67 ± 8 years; 76% were male. Cardiovascular risk factors included hypertension in 77%, hyperlipidemia in 33%, diabetes mellitus in 10%, and current smoking in 36%. During a 5-year follow-up, 32 patients (26%) died and 92 (74%) survived. The annual mortality rate was 5.2%. Patients who died were older than survivors (70 ± 8 vs 66 ± 8 years, *P* = .006), but there were no significant differences in sex, hypertension, hypercholesterolemia, diabetes mellitus, or smoking history. The primary cause of death was cardiovascular in 47% of patients with cancer deaths in 34%. There were no cardiac deaths among patients who had no coronary ischemia (FFR_CT_ > 0.80).

**Table I:** Patient characteristics at baseline in relation to survival status at 5 years

Variables	All N = 124	Alive N = 92	Dead N = 32	*P* value
Age, years	67± 8	66 ± 8	70 ± 8	.006
Male	94 (76)	67 (73)	27 (84)	.189
Female	30 (24)	25 (27)	5 (16)	.189
Hypertension	96 (77)	71 (77)	25 (78)	.912
Hypercholesterolaemia	41 (33)	31 (34)	10 (31)	.800
Diabetes mellitus	12 (10)	7 (8)	5 (16)	.295
Smoking	45 (36)	32 (35)	13 (41)	.554
Antihypertensive drugs	62 (50)	45 (49)	17 (53)	.681
Statins	38 (31)	29 (32)	9 (28)	.720
Insulin	9 (7)	6 (7)	3 (9)	.694
Antiplatelets and/or anticoagulants	56 (45)	38 (41)	18 (56)	.143

Data are presented as n (%) or mean ± standard deviation. *P* values reflect comparisons between survivors (alive) and nonsurvivors (dead) using the independent *t* test for continuous variables and the *χ*^2^ or Fisher exact test for categorical variables.

### Coronary CT-derived plaque and FFR_CT_ analysis

#### Coronary plaque burden

Coronary atherosclerotic plaque burden was high with median TPV 550 mm^3^, interquartile range 299 to 1156, and range 0 to 2684 mm^3^. Plaque characteristics were also high with CPV 97 mm^3^, NCPV 464 mm^3^, and LAPV 9 mm^3^ (Table II). Nonsurvivors had significantly higher plaque volumes across all categories compared with survivors. TPV was nearly twofold higher in nonsurvivors than survivors (975 vs 458 mm^3^; *P* < .001), with similarly higher CPV (229 vs 68 mm^3^; *P* < .001), NCPV (797 vs 383 mm^3^; *P* < .001), and LAPV (12 vs 9 mm^3^; *P* = .038).

**Table II:** Quantitative plaque analysis and coronary ischemia according to survival status

Variables	All N = 124	Alive N = 92	Dead N =32	*P* value
Total plaque volume (TPV), mm^3^	550 [299-1156]	458 [215-906]	975 [499-1419]	<.001
Calcified plaque volume (CPV), mm^3^	97 [38-270]	68 [31-163]	229 [85-375]	<.001
Noncalcified plaque volume (NCPV), mm^3^	464 [246-914]	383 [174-758]	797 [363-1063]	.001
Low attenuation plaque volume (LAPV), mm^3^	9 [3-16]	9 [3-16]	12 [7-19]	.038
Coronary ischemia (FFR_CT_ ≤ 0.80)	66 (53)	44 (48)	22 (69)	.041
Noncoronary ischemia (FFR_CT_ > 0.80)	58 (47)	48 (52)	10 (31)	.041

*FFR*_*CT*_, Computed tomography-derived fractional flow reserve. Data are presented as n (%) or median [interquartile range]. *P* values are derived from the Mann-Whitney *U* test for continuous plaque metrics and the χ^2^ test for coronary ischemia (FFR_CT_**≤** 0.80).

#### Silent coronary ischemia

Asymptomatic (silent) lesion-specific coronary ischemia (FFR_CT_ ≤ 0.80) was present in 66 patients (53%), whereas 47% had no coronary ischemia (Table II). Nonsurvivors had a higher prevalence of silent ischemia than survivors (69% vs 48%; *P* = .041). The 53% prevalence of silent ischemia in this subset is lower than the 69% previously reported in the full lower-extremity revascularization cohort, consistent with exclusion of patients who underwent selective ischemia-targeted coronary revascularization of patients with high-risk ischemia profiles.^18^^,^^19^ Patients with no coronary ischemia were more likely to survive (52% vs 31%; *P* = .041).

#### Stratification according to total plaque volume

**Fig 3:**
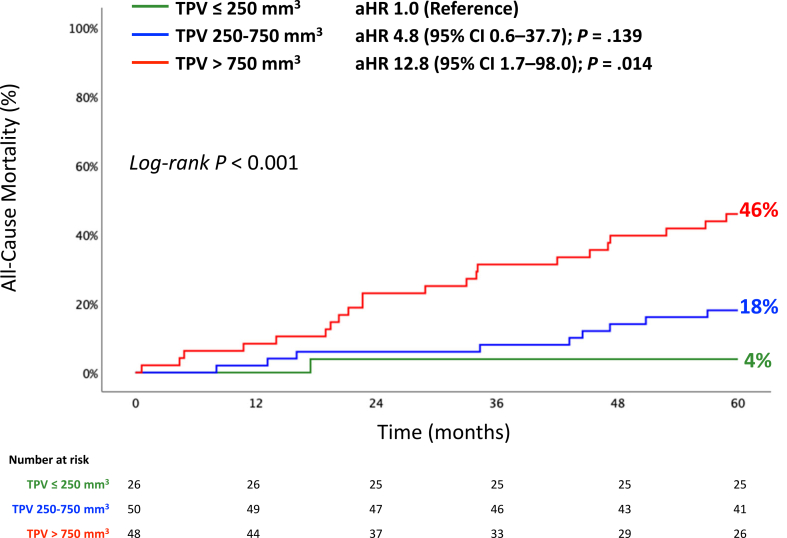
Five-year mortality stratified by total plaque volume (*TPV*). Kaplan-Meier estimates of all-cause mortality stratified by TPV categories of mild (≤250 mm^3^), moderate (251-750 mm^3^), and severe (>750 mm^3^). A dose-response relationship is observed, with a stepwise increase in 5-year mortality (4%, 18%, and 46%, respectively) as plaque burden increases. Mortality is presented as cumulative incidence. Log-rank *P* reflects the overall difference between categories, whereas individual *P* values are derived from the age-adjusted Cox model. *aHR,* Age-adjusted hazard ratio; *CI,* confidence interval.

Stratification of patients by plaque burden categories (TPV: mild ≤ 250 mm^3^; moderate 251-750 mm^3^; and severe > 750 mm^3^) revealed a strong dose-response relationship between plaque volume and mortality (Table III). All-cause mortality at 5 years was 4% in patients with mild plaque, 18% in those with moderate plaque, and 46% in those with severe plaque (log-rank *P* < .001). Compared with patients with mild TPV (≤250 mm^3^), those with severe TPV (>750 mm^3^) had more than a 10-fold increase in risk of death at 5 years (age-adjusted HRs, 12.8; 95% CI, 1.7-98.0; *P* = .014) (Fig 3).

**Table III:** Stratification according to total plaque volume (*TPV*) – mild (≤250 mm^3^), moderate (251-750 mm^3^), or severe (>750 mm^3^)

Variables	TPV ≤ 250 N = 26	TPV 251-750 N = 50	TPV > 750 N = 48	*P* value
Age, years	63 ± 8	65 ± 7	71 ± 8	<.001
Male	20 (79)	37 (74)	37 (77)	.928
Female	6 (23)	13 (26)	11 (23)	.928
Total plaque volume (TPV), mm^3^	114 [52-189]	443 [344-605]	1357 [1012-1708]	<.001
Calcified plaque volume (CPV), mm^3^	12 [1-24]	65 [47-102]	318 [189-459]	<.001
Noncalcified plaque volume (NCPV), mm^3^	98 [50-149]	338 [284-485]	1008 [802-1325]	<.001
Low computed tomographic attenuation plaque volume (LAPV), mm^3^	2 [0-3]	8 [5-12]	17 [12-23]	<.001
Coronary ischemia FFR_CT_ ≤ 0.80	2 (8)	25 (50)	39 (81)	<.001
Five-year mortality	1 (4)	9 (18)	22 (46)	<.001

ANOVA, Analysis of variance; *FFR*_*CT*_, computed tomography-derived fractional flow reserve.

Data are presented as n (%), mean ± standard deviation or median [interquartile range]. *P* values reflect the statistical significance across the three TPV categories using the one-way ANOVA, Kruskal-Wallis test, or χ^2^ test as appropriate.

### Predictors of five-year mortality

In univariate Cox regression analysis, both anatomic and physiologic measures of CAD were significant predictors of 5-year mortality. High TPV (≥550 mm^3^) was associated with a 3.6-fold increased risk of death (HR, 3.6; 95% CI, 1.6-7.9; *P* = .002). The presence of lesion-specific coronary ischemia (FFR_CT_ ≤ 0.80) was associated with a 2.1-fold increased risk of death (HR, 2.1; 95% CI, 1.0-4.5; *P* = .045). After adjusting for age, TPV remained a significant predictor of mortality (adjusted HR, 3.0; 95% CI, 1.3-6.9; *P* = .009), whereas the presence of coronary ischemia was no longer statistically significant (adjusted HR, 1.7; 95% CI, 0.8-3.7; *P* = .169).

### Mortality risk stratified by plaque burden and coronary ischemia

**Fig 4:**
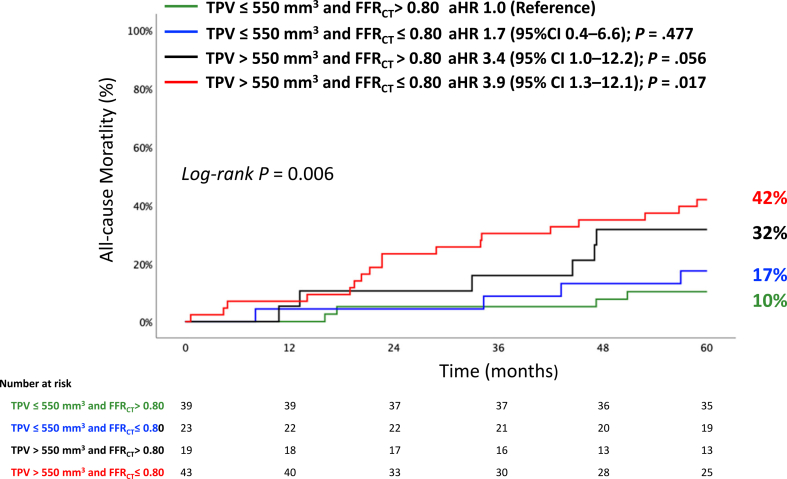
Five-year mortality stratified by total plaque volume (*TPV*) and computed tomography-derived fractional flow reserve (*FFR*_*CT*_). Kaplan-Meier estimates of all-cause mortality stratified by median plaque burden (TPV 550 mm^3^) and presence/absence of lesion-specific ischemia (FFR_CT_ ≤ 0.80). The highest 5-year mortality (42%) was observed in patients with both high-plaque volume and coronary ischemia (*red line*), whereas the lowest mortality risk (10%) was observed in patients with low-plaque volume and no coronary ischemia (*green line*), *P* = .017. Mortality is presented as cumulative incidence. The log-rank *P* value reflects the overall difference between the four risk categories. Individual *P* values and aHRs are derived from the age-adjusted Cox proportional hazards model. *aHR,* Age-adjusted hazard ratio; *CI,* confidence interval.

To explore the combined prognostic value of plaque burden and coronary ischemia, we stratified patients into four groups based on TPV (<550 vs ≥550 mm^3^) and FFR_CT_ (>0.80 vs ≤0.80) (Fig 4). Five-year mortality was 10% in patients with low plaque and no ischemia (TPV < 550 mm^3^ and FFR_CT_ >0.80; reference group), 17% in patients with low plaque and ischemia (TPV <550 mm^3^ and FFR_CT_ ≤ 0.80; age-adjusted HR, 1.7; 95% CI, 0.4-6.6; *P* = .477), 32% in patients with high plaque and no ischemia (TPV ≥ 550 mm^3^ and FFR_CT_ > 0.80; adjusted HR, 3.4; 95% CI, 1.0-12.2; *P* = .056), and 42% in patients with high plaque and ischemia (TPV ≥550 mm^3^ and FFR_CT_ ≤0.80; adjusted HR, 3.9; 95% CI, 1.3-12.1; *P* = .017). Thus, the combination of high plaque burden and coronary ischemia identified a very high-risk group with 42% 5-year mortality—more than fourfold higher than patients with low plaque burden and no ischemia (log-rank *P* = .006).

## Discussion

This study provides the first evidence that quantitative coronary plaque burden and FFR_CT_-derived ischemia can predict 5-year mortality in patients with PAD with asymptomatic CAD undergoing major vascular surgery and treated with medical therapy alone. Despite the absence of cardiac symptoms or known CAD and exclusion of patients who underwent ischemia-guided coronary revascularization, these patients had a substantial burden of coronary plaque (median TPV, 550 mm^3^) and a high prevalence of silent coronary ischemia (53%). Although there were no perioperative deaths, these coronary findings had a profound impact on long-term survival: patients with high coronary plaque volume and silent ischemia had a fourfold higher risk of death at 5 years compared with patients with low-plaque volume and no ischemia (42% vs 10%). These results demonstrate that coronary CTA with CT-QPA and FFR_CT_ can effectively risk-stratify patients with PAD and identify both high- and low-risk groups for the long-term mortality.

The overall plaque burden in this cohort (median TPV, 550 mm^3^) is higher than that reported in large symptomatic CAD cohorts undergoing clinically indicated CTA, where median TPV is typically in the range from 200 to 300 mm^3^.^14^^,^^15^ This is consistent with the known higher mortality of patients with PAD compared with typical CAD populations.^1^^,^^2^^,^^9^^,^^22^^,^^24^ Coronary plaque burden was found to be a strong predictor of 5-year survival: in nonsurvivors, TPV was approximately twofold higher than in survivors (975 vs 458 mm^3^; *P* < .001), with similar increases in calcified and non-CPVs. In multivariate analysis, high TPV (≥550 mm^3^) was independently associated with mortality (adjusted HR, 3.0; 95% CI, 1.3-6.9; *P* = .009), whereas severe plaque burden (TPV > 750 mm^3^) conferred a 12-fold higher risk of death (*P* = .014). This dose-response relationship between plaque burden and mortality is well established in patients with symptomatic CAD^13^, ^14^, ^15^, ^16^, ^17^ but has not previously been demonstrated in asymptomatic patients with PAD.

FFR_CT_ assessment identified silent coronary ischemia in 53% of patients, and this was associated with a twofold higher mortality in univariate analysis (HR, 2.1; 95% CI, 1.0-4.5; *P* = .045). However, multivariate analysis did not show ischemia as an independent predictor after adjusting for age and plaque burden, likely due to the exclusion of higher-risk patients who underwent ischemia-guided coronary revascularization and the limited number of events in our cohort. Silent ischemia is a well-known marker of high risk for sudden cardiac death and acute MI,^25^ and the lower prevalence of ischemia in this subset compared with the full lower-extremity revascularization cohort (53% vs 69%) is consistent with clinical triage of patients with more extensive ischemia to revascularization. Clinically, the most robust signal came from the combined risk-group analysis: patients with both high-plaque burden and coronary ischemia had 42% 5-year mortality, whereas those with low-plaque burden and no ischemia had only 10% mortality (age-adjusted HR, 3.9; *P* = .017), representing a clinically meaningful risk gradient that could help guide management decisions.

Clinical reports in PAD populations have focused primarily on clinical risk factors, procedural outcomes, and postoperative MI, with limited data on the role of coronary anatomy and physiology on long-term outcomes. We have previously shown that systematic diagnosis of silent coronary ischemia using CTA and FFR_CT_ together with selective coronary revascularization can reduce all-cause death, cardiovascular death, and MI by more than 50% compared with current guideline-directed medical management.^18^^,^^19^ The current analysis extends those findings by showing that, among medically managed patients who did not undergo coronary revascularization, quantitative plaque burden and FFR_CT_ remain powerful predictors of long-term mortality. This underscores the value of coronary CTA-based risk stratification even when revascularization is not performed.

These observations parallel findings from symptomatic CAD populations in which CTA, CT-QPA, and FFR_CT_ have been integrated into guideline-directed evaluation and management.^12^ In the International Study od Comparative Health Effectiveness with Medical and Invasive Approaches (ISCHEMIA) trial, TPV was one of the strongest predictors of cardiovascular death or MI among patients with moderate-to-severe ischemia.^13^ The Prospective Multicenter Imaging Study for Evaluation of Chest Pain (PROMISE) trial demonstrated that high-risk plaque features on CTA improve risk stratification beyond clinical variables and stenosis severity.^15^ The FFRCT in Stable Heart diseae & CTA Helps Improve Patient care and Societal costs (FISH&CHIPS) study in nearly 8000 patients showed that TPV was a powerful independent predictor of cardiovascular death and MI during long-term follow-up with patients in the highest plaque category (>750 mm^3^) had a fivefold increased risk compared with low TPV.^26^ In our study, the risk of death at 5 years was 12-fold higher in patients with PAD with TPV > 750 mm^3^). The prognostic value of FFR_CT_ has been shown in the Assessing Diagnostic Value of Non-invasive FFRCT in Coronary Care - Danish subset (ADVANCE-DK) registry of 900 patients with stable angina with a 3.3-fold increased risk in death and MI with FFR_CT_ ≤ 0.80 at 3-year follow-up^16^ with continued benefit extending to 7 years.^17^ Our results extend these concepts to asymptomatic patients with PAD with no known CAD, a group largely excluded from previous coronary trials and in whom current PAD guidelines do not recommend routine cardiac testing.^5^^,^^6^

The high-risk and poor long-term survival of patients with PAD is well documented with key predictors of death identified as age, CAD, chronic kidney disease, diabetes mellitus, low-functional capacity, and frailty. However, there is little information about “good risk” patients who also have poor long-term survival following vascular surgery procedures. Patients in the present study represent a low-risk subset of a high-risk PAD population: they were relatively young (mean age, 67 years), had no known CAD or chronic kidney disease, and only 10% had diabetes. Furthermore, higher-risk patients with significant coronary ischemia who underwent coronary revascularization were excluded from this current study leaving an even lower-risk population. As a result, 5-year mortality in our cohort (26%) was lower than that reported in randomized trials such as BEST-CLI and BASIL-2 and in real-world registries such as SAFE-PAD and VQI-VISION which show 5-year mortality of 50% to 70% in patients with chronic limb-threatening ischemia.^1^^,^^2^^,^^8^^,^^27^ Nevertheless, plaque burden and combined plaque-ischemia staging still provided substantial prognostic discrimination, underscoring their utility even after higher-risk patients are triaged to revascularization.

### Clinical implications

Our findings have important implications for the management of patients with PAD undergoing elective vascular surgery. Current PAD and noncardiac surgery guidelines recommend against routine preoperative cardiac testing in patients with PAD without cardiac symptoms, based largely on older studies that used angiographic stenosis severity rather than ischemia-guided strategies.^28^ In contrast, the 2021 American Heart Association/American College of Cardiology chest pain guideline emphasizes coronary CTA as the frontline diagnostic test and advocates plaque analysis to guide medical therapy and FFR_CT_ to guide decisions regarding coronary revascularization in patients with 40% to 90% stenosis.^10^ It should be noted that coronary CTA requires contrast injection to reveal coronary anatomy and physiology and should not be confused with the commonly used calcium score, which provides only a risk score with no specific information about the coronary arteries. Our study using coronary CTA shows that even in young patients with PAD with no known CAD, who are considered to be “low risk,” more than half harbor silent ischemia with substantial plaque burden. At the same time, some have little coronary plaque and no ischemia, which portends good long-term survival. Routine cardiac testing of patients with PAD using coronary CTA with coronary plaque and FFR ischemia testing may risk-stratify patients and help guide decisions regarding management of CAD to reduce adverse cardiac events and improve long-term survival. Patients with high-plaque burden and ischemia (42% 5-year mortality) may benefit from more intensive surveillance, optimization of guideline-directed medical therapy, and consideration of coronary revascularization, whereas patients with low-plaque burden and no ischemia (10% 5-year mortality) may reasonably be managed with medical therapy alone.

Coronary CTA with CT-QPA and FFR_CT_ are diagnostic modalities which are cleared by the Food and Drug Administration, covered by Medicare and most private insurance, and readily available in most health care institutions that care for patients presenting with suspected CAD.

### Limitations

This study is limited by its single-center, real-world observational design with a selection bias towards low-risk patients, which may not reflect the broader population of vascular surgery patients. The study population was skewed by the exclusion of patients who underwent ischemia-guided coronary revascularization, thus reducing the risk profile and mortality of patients included in this analysis. This may limit the generalizability to patients with more severe or symptomatic CAD. However, this selection was intentional and clinically appropriate, as it allows assessment of the natural prognostic value of plaque burden in patients managed with guideline-directed medical therapy—the current standard of care for patients with PAD with asymptomatic CAD. Furthermore, the modest sample size (n = 124) with 32 deaths limits the power of multivariable analyses and yields wide CIs for some estimates, particularly for severe plaque burden (TPV > 750 mm^3^) indicating uncertainty regarding the exact magnitude of risk. In addition, the study was not designed to test whether CTA-guided interventions improve survival; rather, it evaluated the prognostic significance of CT-QPA and FFR_CT_ in medically treated patients. External validation in independent cohorts is needed to establish optimal plaque and ischemia thresholds for clinical decision-making in patients with PAD. Finally, randomized controlled trials are required to determine whether coronary CTA-guided management of patients with PAD with asymptomatic CAD undergoing major vascular surgery improves long-term survival compared with current guideline-directed medical therapy alone.

## Conclusions

In patients with PAD with no cardiac symptoms undergoing elective vascular surgery, coronary plaque burden quantified by CT-QPA and silent ischemia detected by FFR_CT_ are strong predictors of 5-year mortality. Patients with high-plaque burden and coronary ischemia have a 42% 5-year mortality, compared with 10% in those with low-plaque burden and no ischemia. Coronary CTA with quantitative plaque and FFR analysis should be considered for comprehensive cardiac risk stratification in vascular surgery patients, regardless of cardiac symptoms, and randomized trials are needed to define the role of CTA-guided risk stratification and treatment in patients with PAD with asymptomatic CAD.

## Author Contributions

Conception and design: DK, SJ, EZ, GL, IK, AE, CZ

Analysis and interpretation: DK, SJ, FA, EZ, GL, IK, DP, LZ, AE, CZ

Data collection: DK, SJ, AL, EZ, GL, IK, DP, JV, ES, LZ, LT, LL, AE, CZ

Writing the article: DK, EZ, CZ

Critical revision of the article: DK, SJ, FA, AL, EZ, GL, IK, DP, JV, ES, LZ, LT, LL, AE, CZ

Final approval of the article: DK, SJ, FA, AL, EZ, GL, IK, DP, JV, ES, LZ, LT, LL, AE, CZ

Statistical analysis: SJ, GL, LT

Obtained funding: DK

Overall responsibility: DK

## Funding

HeartFlow Inc has provided institutional support to Pauls Stradins Clinical University Hospital for CTA and FFRCT analysis. The Latvian Council of Science (Project No. 2018/2-0295) and the Ministry of Education and Science (Project No. 2023/3-0004) partially funded this research. None of the supporters influenced study design, data collection, analysis or interpretation of data, manuscript writing, the decision to submit the manuscript for publication or any other involvement in the creation of the manuscript.

## Disclosures

No authors have a potential conflict of interest regarding the content herein with the exception of C.K.Z. who has a financial interest in HeartFlow, Inc.
